# Electrical behaviour of native cellulose nanofibril/carbon nanotube hybrid aerogels under cyclic compression†
†Electronic supplementary information (ESI) available. See DOI: 10.1039/c6ra16202a



**DOI:** 10.1039/c6ra16202a

**Published:** 2016-09-05

**Authors:** Miao Wang, Ilya V. Anoshkin, Albert G. Nasibulin, Robin H. A. Ras, Nonappa, Janne Laine, Esko I. Kauppinen, Olli Ikkala

**Affiliations:** a Department of Applied Physics , School of Science , Aalto University , P. O. Box 15100 , FI-00076 Espoo , Finland . Email: olli.ikkala@aalto.fi; b Skolkovo Insititute of Science and Technology , Nobel str. 3 , Moscow , 143026 , Russia; c Saint-Petersburg State Polytechnical University , Department of Material Science , Polytechnicheskaya 29 , 195251 , Saint-Petersburg , Russia; d Department of Forest Products Technology , School of Chemical Technology , Aalto University , P. O. Box 16300 , FI-00076, Espoo , Finland

## Abstract

Hybrid aerogels consisting of cellulose nanofibers (CNF) and modified few-walled carbon nanotubes (FWCNT) are investigated under cyclic mechanical compression to explore “electrical fatigue”. For this purpose the FWCNTs were hydrophilized, thus promoting their aqueous dispersibility to allow FWCNT/CNF hybrid hydrogels, followed by freeze-drying to obtain hybrid aerogels. The optimized composition consisting of FWCNT/CNF 20/80 wt/wt showed conductivity of 10^–5^ S cm^–1^ as promoted due to double percolation, and showed only small changes in electrical and mechanical behaviour upon cycling 100 times. The electrical behaviour under cycled compression shows good stability and reversibility.

## Abstract 2

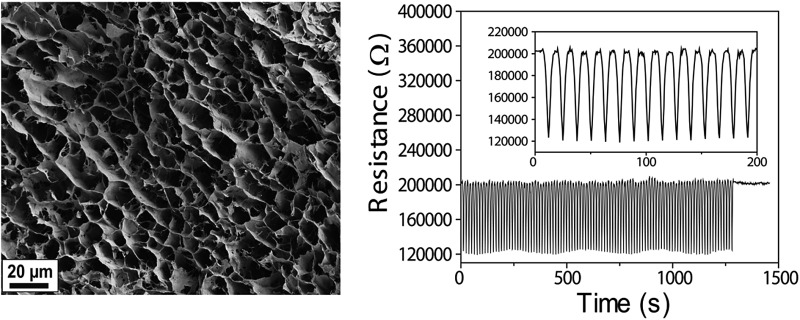
Hybrid aerogels consisting of cellulose nanofibers (CNF) and modified few-walled carbon nanotubes (FWCNT) are investigated under cyclic mechanical compression to explore “electrical fatigue”.

## Introduction

Highly porous and lightweight native nanocellulose aerogels or sponge-like materials have aroused extensive interest.^1–33^ Unlike classic aerogels based on silica, which are relatively brittle, they can allow reduced brittleness and even flexibility, combining very low densities and high porosities.^2–4,7,34,35^ Cellulose nanofibrils (CNF), also known as nanofibrillar cellulose (NFC) or microfibrillated cellulose (MFC), are attractive due to the enhanced mechanical properties of native cellulose crystals with a modulus of *ca.* 140 GPa,^36^ and the fact that cellulose is a sustainable and the most abundant polymer on Earth as it is available from plant cell walls.^37^ Importantly, to preserve the strong native crystalline form, specific processes have been developed to cleave without dissolution the 3–20 nm diameter nanofibers from plant cell walls in practical ways.^38–40^ CNF aerogels are typically produced from CNF hydrogels by removing water upon freeze-drying or supercritical drying, leading to interconnected sheet-like or fibrous skeleton respectively, depending on the process.^9^ Thus, lightweight and flexible aerogels with long cellulose I nanofibers have been produced. This has allowed even ultra-low density aerogels (down to 0.2 × 10^–3^ g cm^–3^) and porosity as high as 98%.^13^ The high porosity and mechanically strong CNF aerogel allows variety of applications in filtration,^41^ insulation^31,42^ and as templates for functionalities.^2,9–11,14,19,30^ Conducting CNF aerogels have been made by functionalizing with conjugated polymers^2,43–45^ or by mixing with carbon nanotubes.^19,25^ Recently a robust and rapid method has been shown for the layer-by-layer assembly of functional polymers and nanoparticles on cross-linked nanocellulose aerogels with a porosity close to 99%, high strength, and nanoscale shape integrity in water.^22^ However, there still exist challenges to improve the mechanical properties of CNF aerogels in comparison to the natural porous material, *e.g.* wood, bone and tendon.^46^ Recently, nanocellulose composite biofoams have been made with high mechanical performance.^3,6,47^ A biomimetic polysaccharide foams were prepared by the lyophilization technique with cellulose nanofibril content ranging from 10 to 70 wt% (dry weight basis).^3^ Compared to the neat amylopectin foam, a significant improvement in modulus and yield strength was observed. The control of ice crystal growth in freezing was critical to tailor the aerogel morphology.

**Fig. 1:**
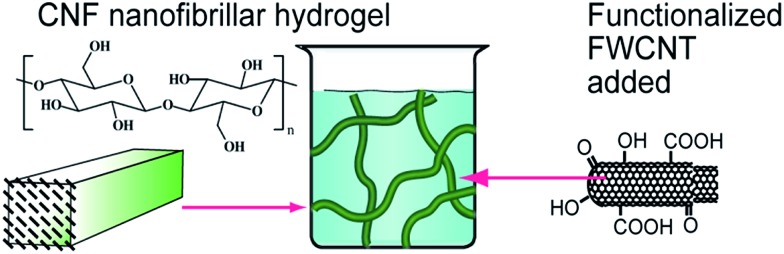
Schematic of blending of native cellulose nanofibrils and functionalized few-walled carbon nanotubes to hybrid hydrogels, which are further freeze-dried for hybrid aerogels.

Previously, we have demonstrated mechanoresponsive conductivity and pressure sensing materials by modifying CNF aerogels with modified few-walled carbon nanotubes (FWCNT).^19^ Both components are 1-dimensional nanoscale objects with high mechanical properties. The hybrid FWCNT/CNF aerogels were shown to be ductile in isolated compressions, and allowed systematic resistance change as a function of deformation.^19^ In this work we explore the electrical stability of the FWCNT/CNF aerogels under repeated compression cycles, *i.e.* “electrical fatigue” (see Fig. 1 for the components). This is a nontrivial aspect, depending on the subtle details of robustness of percolations of FWCNT within the hybrid aerogel. We explore the structural properties using electron microscopy, as well as the electrical and mechanical properties under repeated compressional cycles.

## Experimental section

### Preparation of water dispersible FWCNT

FWCNTs were produced by catalytic pyrolysis of CH_4_ in the presence of H_2_ (20/80 v/v) at 960 °C on the (Co–Mo)/MgO catalyst.^48^ The FWCNTs were purified with HCl (35 wt%) to remove the residual catalyst and they were annealed at 380–390 °C in an air atmosphere to eliminate the amorphous carbon. In order to promote dispersibility in water, FWCNTs were functionalized by oxygen containing groups (mostly –COOH, >C=O and –OH), using a mixture of concentrated nitric acid (70 wt%) and concentrated sulfuric acid (96 wt%) with a volume ratio of 1 : 3 at 80 °C for 3–4 hours under stirring using a method described before.^49^ The functionalized FWCNTs were then iteratively washed on a filter by acetone until clean and colourless filtrate was obtained in order to separate the non tubular reaction products. Thereafter the functionalized FWCNTs were washed by deionized water and 5% HCl until the sulfate ions could not be detected in the infiltrated water using reaction with barium ions. The solution was then freeze-dried and finally powders were received. In this work, subsequently the denotion FWCNT refers to the above modified materials.

### Preparation of FWCNT/CNF aqueous mixtures

The functionalized dry FWCNTs powders were added in deionized water at concentration 2 wt% and mixed using ultrasound tip (Branson Digital Sonifier, Model 450-D) at 30% amplitude and 15 min with pulse on 2 s and off 1 s. Different amounts of 2 wt% FWCNTs dispersions were added to CNF hydrogels to render the FWCNT loading at 5, 10, 15, 20, and 25 wt/wt in the final FWCNT/CNF aerogels, and also to ensure the dry content of whole mixture for all loadings around 2 wt% which will make the dry aerogels had similar density around 0.02 g cm^–3^. An Ultra-Turrax (IKA, T25 Basic) was used for mixing at 11 000 rpm for 2 min. Subsequently, all mixtures were kept in the ultrasonic bath (Transsonic T420, Elma GmbH & Co KG, Germany) for 15 min.

### FWCNT/CNF hybrid aerogel preparation

A silicone embedding mold was used (Electron Microscopy Science, cavity 14 × 7 × 4 mm) to make aerogels by freeze-drying in liquid nitrogen (*ca.* –196 °C). The mold was inserted to liquid nitrogen at a constant controlled rate of 6.6 mm min^–1^. The subsequent ice sublimation was performed with a Labconco freeze dryer system (FreeZone –105 °C 4.5 liter cascade benchtop freeze dryer systems, Labconco, U.S.A). Most feasible property is shown by the FWCNT/CNF 20/80 wt/wt compositions, which will be in the main emphasis of this work.

### Field-emission scanning electron microscopy (FE-SEM)

Field-emission scanning electron microscopy (Zeiss Sigma VP, U.S.A) was performed at 1.5 keV electron energy. All the samples were sputtered with a thin layer of gold (Emitech K950X/K350) prior to imaging.

### Transmission electron microscopy (TEM)

The transmission electron microscopy (TEM) images were collected using JEM 3200FSC field emission microscope (JEOL) operated at 300 kV in bright field mode with Omega-type zero-loss energy filter. The images were acquired with Gatan digital micrograph software while the specimen temperature was maintained at –187 °C. The TEM sample of FWCNTs was prepared by dropcasting 3 μL of the sample over a plasma cleaned quantifoil 400 mesh grid with holey carbon. The aerogel samples were placed on a Oyster 300 mesh foldable grid and imaged.

### Conductivity

Conductivity was measured with a Keithley 2400 source meter using two-wire mode. Samples were connected with copper wires in two ends at the length direction using conductive silver glue to optimize the electric contact. Same set up was used also for compression sensing tests.

### Cyclic and compression sensing testing

Cyclic compression test was implemented with a 1 kN load cell Instron testing machine (Instron Universal Materials Testing Machine 5567, Instron, Norwood, MA). The cyclic testing was set to 8% compression strain at 3 mm min^–1^ rate loading and unloading. Samples were connected with copper wires in two ends using conductive silver glue. Resistance was detected meanwhile with a Keithley 2400 source meter and KickStart software. Both the compression clamps were insulated by Kapton tape. All testing was made at room temperature at around 25 ± 5% relative humidity.

## Results and discussion

### Morphology of FWCNT/CNF hybrid aerogels

**Fig. 2:**
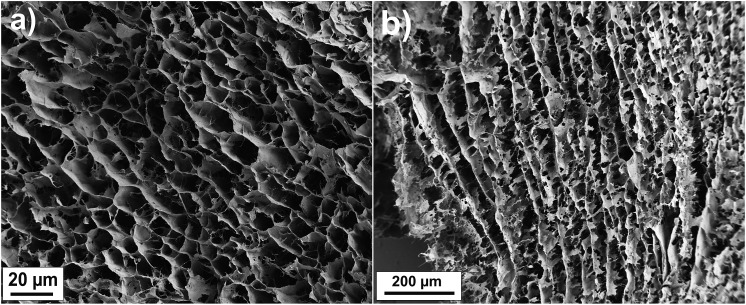
Scanning electron microscopy images of FWCNT/CNF 20/80 wt/wt hybrid aerogel top (a) and side (b) view, showing only a small anisotropy involving a porous skeleton structure in all directions.

**Fig. 3:**
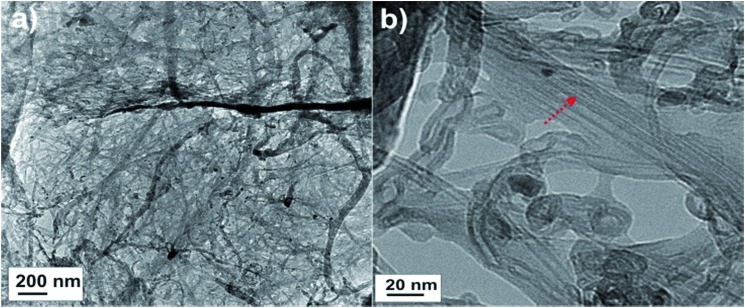
Transmission electron microscopy images of FWCNT/CNF 20/80 wt/wt hybrid aerogel in low (a) and high (b) magnification. The red arrow points out carbon nanotubes in the aerogel network.

Visual inspection suggests good dispersibility of the functionalized FWCNTs in water at concentration 2 wt%, as promoted by the oxygen containing hydrophilic groups on their surfaces (Fig. 1). Based on TEM, individualized FWCNTs have diameter around 3.2 nm, however, also bundles are observed (Fig. S1†). That the present CNFs dispersed well in aqueous medium is well known in the previous literature; see TEM micrographs of similar CNFs shown in *e.g.* ref. 19. The morphology of the freeze-dried hybrid FWCNT/CNF aerogel was inspected using SEM. Most emphasis will subsequently be given to the composition FWCNT/CNF 20/80 wt/wt, as this composition will turn most promising under repeated cycles, as will be discussed later. As the used freezing-drying method with controlled immersion speed into liquid nitrogen (see Experimental section) could, in principle, lead to anisotropies in the aerogel porosity, we investigated the morphology in the top and side views (Fig. 2a and b and S2†). From above, *i.e.* perpendicular to the ice-growth direction, SEM suggests honeycomb-like structures (Fig. 2a). The side-view, along the ice-growth front, indicates only slight anisotropy with tubular formation with skeleton-like tube walls (Fig. 2b). One can conclude that the aerogel structure is essentially isotropic as it involves in each direction a porous skeleton structure. The findings can be compared to previous studies, which showed macroporous honeycomb structures with continuous tubular walls based on multiwalled carbon nanotube aerogels taken they involve an additional polymer phase as a crosslinking component.^50^ In this case, it is much easier to form continuous honeycomb tube walls using the continuous polymer phase, in comparison to the present situation involving two 1-dimensional nano-objects. TEM was incorporated to investigate the structures in the nanoscale (Fig. 3a and b). Composite fibrillar network of the FWCNT/CNF is inferred in Fig. 3a where FWCNTs are incorporated within the CNF network forming the majority phase in FWCNT/CNF 20/80 wt/wt aerogels.

### Conductivity

**Fig. 4:**
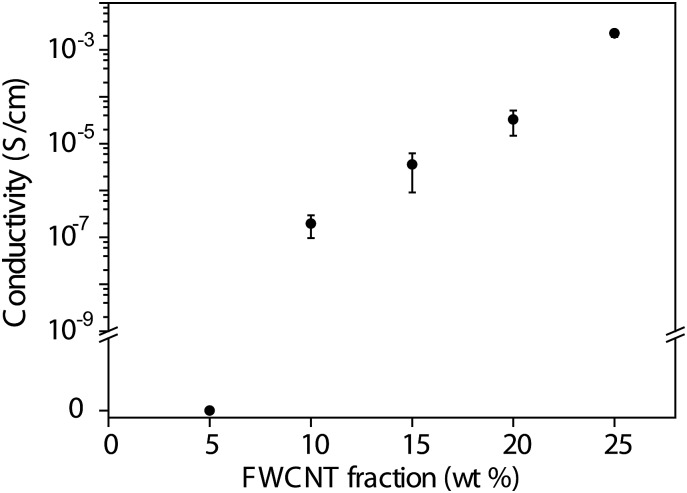
Electrical conductivity as a function of carbon nanotube weight fraction in FWCNT/CNF hybrid aerogels. Note that due to the highly porous structure of the aerogel, the volumetric percolation threshold is very low, a fraction of % vol.

Conductivity is shown in Fig. 4 as a function of FWCNT weight fraction in the hybrid aerogels. Below 10 wt% FWCNT loading, no conductivity is seen in the material. In this case the carbon nanotubes do not form a percolative network within CNF skeleton. Upon increasing the FWCNT loading from 10 wt% to 25 wt%, the conductivity increases 4 orders of magnitude, reaching the conductivity 2 × 10^–3^ S cm^–1^. We suggest that, in fact, this involves double percolation, where first the CNF skeleton percolates in the geometric volume of the aerogel, and then the FWCNT percolates within the CNF skeleton.

### Humidity effect

**Fig. 5:**
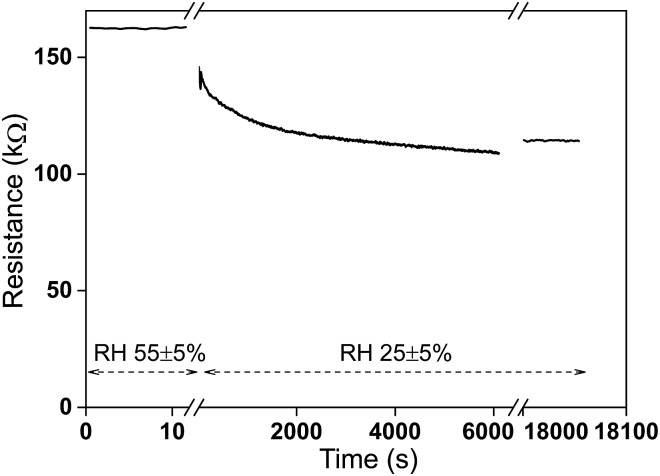
Resistance as a function of time between two different humidity environments. A FWCNT/CNF 20/80 wt/wt aerogel was kept in 55 ± 5% relative humidity inside a humidity chamber overnight and taken out immediately in the same room with 25 ± 5% relative humidity to check resistance change for two hours and after five hours.

CNFs are hydrophilic because of the abundant hydroxyl groups leading to extensive water vapour adsorption. The FWCNT used in this work also involves considerable amount of oxygen-containing groups, which will also make them hydrophilic. In order to gain some understanding how the water vapour influences the resistance level of the present hybrid aerogels, the resistance of the aerogel is investigated between different humidity environments. Fig. 5 illustrates the behaviour where FWCNT/CNF 20/80 wt/wt aerogel was first equilibrated overnight at a high humidity (55 ± 5% RH) in a humidity chamber and then taken out to lower humidity (25 ± 5% RH). Thereupon the resistance decreases *ca.* 30% and starts to approach an equilibrium in two hours. After five hours the resistance level maintains almost same value as after the first two hours. The conclusion is that humidity has a large effect on the resistance. When practical applications are pursued, the aerogel has to be functionalized by hydrophobizations. There are several ways described in the literatures.^10,11,14^ In the subsequent cyclic compression tests the humidity is fixed to 25 ± 5% RH at room temperature.

### Cyclic compression tests: electrical and mechanical behaviour

**Fig. 6:**
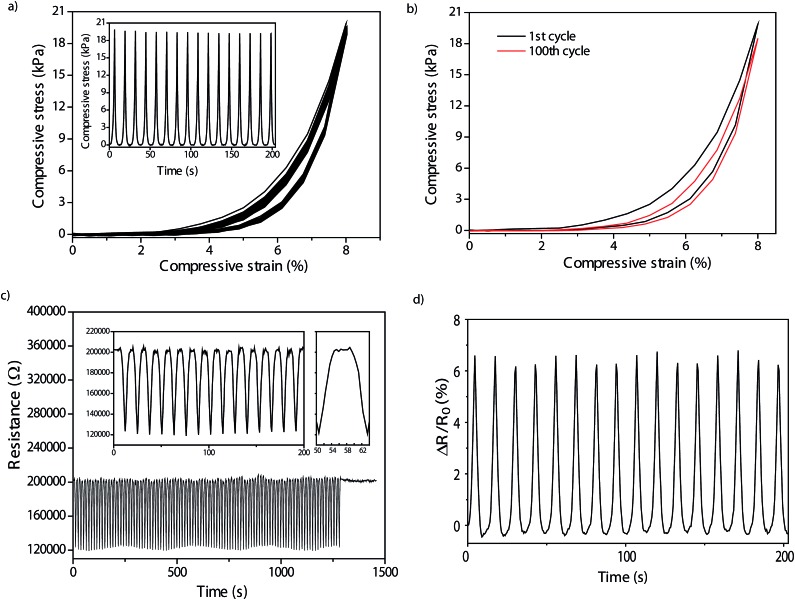
Cyclic mechanical and electrical compression studies for the FWCNT/CNF 20/80 wt/wt hybrid aerogel. (a) 100 cycled stress–strain curves up to strain 8% and the 16 first compressive stress *vs.* time cycles illustrated (inset). (b) The first and 100^th^ cycle of compressive stress–strain curves. (c) 100 cycled resistance response *vs.* time under cyclic compression up to strain 8%. The insets show the 16 first cycles, and one characteristic cycle lasting 12 seconds. (d) The fractional resistance reduction during cyclic compression.

In this work we selected a strain level of 8% for cyclic compressions, which is expected to be in the linear regime. Also, previous studies have demonstrated the cellular nanofibrillar cellulose foam has an initial linear elastic regime up to a strain of about 12%.^46^ Fig. 6a illustrates 100 cyclic stress–strain curves for the FWCNT/CNF 20/80 wt/wt aerogel and, for clarity, Fig. 6b highlights the extracted 1^st^ and 100^th^ curve. Thus, a reasonably reversible compression cyclability is shown for 100 cycles, where there is only a small change from the first cycle to the last cycle observed. It also indirectly supports that 8% strain is in the elastic region as the material recovers upon unloading. The hysteresis between loading and unloading is relatively small for FWCNT/CNF 20/80 wt/wt aerogel. By contrast, in the first cycle of FWCNT/CNF 25/75 wt/wt aerogel, the hysteresis is bigger (Fig. S3b†). But after 100^th^ cycle, the hysteresis becomes reduced. In general, hysteresis can be reduced by applying lower strains.^51^

**Fig. 7:**
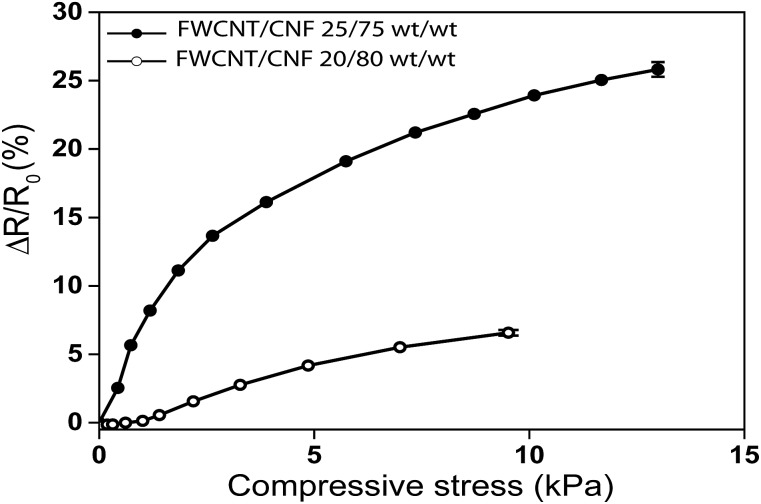
The fractional resistance reduction (Δ*R*/*R*_0_) as a function of imposed compressive stress for different FWCNT loadings. At small loading 15 wt% the conductivity is too low to render a reproducible behaviour. 20 wt% gives the most reversible behaviour in both electrical and mechanical terms and 25 wt% indicates a higher fractional resistance reduction, but starts to show irreversibility in mechanical behaviour, whereas 25 wt% is highly irreversible.

The resistances and fractional resistance reductions during the cyclic compression are illustrated in Fig. 6c and d. Resistance shows almost immediate response corresponding to compressive loading and unloading. Each cycle takes *ca.* 12 seconds (see inset of Fig. 6c). The fractional resistance reduction (Δ*R*/*R*_0_) for FWCNT/CNF 20/80 wt/wt changes reversibly *ca.* 7%, and it is essentially similar after 100 cycles (Fig. 6d). This indicates that the electrical properties in cycling of the aerogel materials show good stability for such a composition. Fig. 7 shows the reversible fractional resistance reduction as a function of compressive stress for both FWCNT/CNF 20/80 wt/wt and 25/75 wt/wt, showing that for the latter aerogel the absolute resistance changes even 25% and also it stays approximately stable for 100 cycles (Fig. S3†). But for that composition involving higher FWCNT fraction the mechanical cyclability was not as stable as for 20/80 wt/wt. As a comparison, the FWCNT/CNF 15/85 wt/wt aerogel cyclic behaviour is inferior in comparison to the above-mentioned aerogels (Fig. S3†) in which the resistance level gradually decreases upon loading and unloading. We assign this problem to the low conductivity value, which is close to percolation threshold.

## Conclusions

In conclusion, we have shown that FWCNT/CNF hybrid aerogels can be constructed to allow stable reversible resistance reduction in cyclic compression. Composition involving FWCNT/CNF 20/80 wt/wt was observed to be promising, showing conductivity of 10^–5^ S cm^–1^ obviously as facilitated by the double percolation, where first CNF percolates in the aerogel geometric volume and then FWCNT percolates within the latter skeleton. In cyclic compressions this aerogel showed reversible resistance change, and only small changes were observed during 100 cycles. At small FWCNT loading (FWCNT/CNF 15/85 wt/wt) the conductivity is too low to render a reproducible behaviour. FWCNT/CNF 20/80 wt/wt gives the most reversible behaviour in both electrical and mechanical terms, whereas FWCNT/CNF 25/75 wt/wt shows higher fractional resistance reduction, but starts to show irreversibility in mechanical behaviour. We foresee that combining cellulose nanofibrils and carbon nanotubes and controlling the morphology allows a wide variety of responsive electroactivity, pressure sensing and functional materials.

## References

[cit1] Capadona J. R., van den Berg O., Capadona L. A., Schroeter M., Rowan S. J., Tyler D. J., Weder C. (2007). Nat. Nanotechnol..

[cit2] Pääkkö M., Vapaavuori J., Silvennoinen R., Kosonen H., Ankerfors M., Lindström T., Berglund L. A., Ikkala O. (2008). Soft Matter.

[cit3] Svagan A. J., Samir M. A. S. A., Berglund L. A. (2008). Adv. Mater..

[cit4] Sehaqui H., Salajkova M., Zhou Q., Berglund L. A. (2010). Soft Matter.

[cit5] Aulin C., Netrval J., Wågberg L., Lindström T. (2010). Soft Matter.

[cit6] Svagan A. J., Jensen P., Dvinskikh S. V., Furo I., Berglund L. A. (2010). J. Mater. Chem..

[cit7] Olsson R. T., Azizi Samir M. A. S., Salazar Alvarez G., Belova L., Strom V., Berglund L. A., Ikkala O., Nogues J., Gedde U. W. (2010). Nat. Nanotechnol..

[cit8] Heath L., Thielemans W. (2010). Green Chem..

[cit9] Korhonen J. T., Hiekkataipale P., Malm J., Karppinen M., Ikkala O., Ras R. H. A. (2011). ACS Nano.

[cit10] Korhonen J. T., Kettunen M., Ras R. H. A., Ikkala O. (2011). ACS Appl. Mater. Interfaces.

[cit11] Jin H., Kettunen M., Laiho A., Pynnönen H., Paltakari J., Marmur A., Ikkala O., Ras R. H. A. (2011). Langmuir.

[cit12] Saito T., Uematsu T., Kimura S., Enomae T., Isogai A. (2011). Soft Matter.

[cit13] Chen W., Yu H., Li Q., Liu Y., Lia J. (2011). Soft Matter.

[cit14] Kettunen M., Silvennoinen R. J., Houbenov N., Nykänen A., Ruokolainen J., Sainio J., Pore V., Kemell M., Ankerfors M., Lindström T., Ritala M., Ras R. H. A., Ikkala O. (2011). Adv. Funct. Mater..

[cit15] Gebald C., Wurzbacher J. A., Tingaut P., Zimmermann T., Steinfeld A. (2011). Environ. Sci. Technol..

[cit16] Liu S., Yan Q., Tao D., Yu T., Liu X. (2012). Carbohydr. Polym..

[cit17] Carlsson D. O., Nyström G., Zhou Q., Berglund L. A., Nyholm L., Stromme M. (2012). J. Mater. Chem..

[cit18] Han J., Zhou C., Wu Y., Liu F., Wu Q. (2013). Biomacromolecules.

[cit19] Wang M., Anoshkin I. V., Nasibulin A. G., Korhonen J. T., Seitsonen J., Pere J., Kauppinen E. I., Ras R. H. A., Ikkala O. (2013). Adv. Mater..

[cit20] Javadi A., Zheng Q., Payen F., Javadi A., Altin Y., Cai Z., Sabo R., Gong S. (2013). ACS Appl. Mater. Interfaces.

[cit21] Gao K., Shao Z., Wang X., Zhang Y., Wang W., Wang F. (2013). RSC Adv..

[cit22] Hamedi M., Karabulut E., Marais A., Herland A., Nyström G., Wågberg L. (2013). Angew. Chem., Int. Ed..

[cit23] Melone L., Altomare L., Alfieri I., Lorenzi A., De Nardo L., Punta C. (2013). J. Photochem. Photobiol., A.

[cit24] Li W., Zhao X., Liu S. (2013). Carbohydr. Polym..

[cit25] Hamedi M. M., Hajian A., Fall A. B., Håkansson K., Salajkova M., Lundell F., Wågberg L., Berglund L. A. (2014). ACS Nano.

[cit26] Wang Y., Yadav S., Heinlein T., Konjik V., Breitzke H., Buntkowsky G., Schneider J. J., Zhang K. (2014). RSC Adv..

[cit27] Wang P., Zhao J., Xuan R., Wang Y., Zou C., Zhang Z., Wan Y., Xu Y. (2014). Dalton Trans..

[cit28] Jiang F., Hsieh Y.-L. (2014). J. Mater. Chem. A.

[cit29] Zhang Z., Sèbe G., Rentsch D., Zimmermann T., Tingaut P. (2014). Chem. Mater..

[cit30] Toivonen M. S., Kaskela A., Rojas O. J., Kauppinen E. I., Ikkala O. (2015). Adv. Funct. Mater..

[cit31] Wicklein B., Kocjan A., Salazar-Alvarez G., Carosio F., Camino G., Antonietti M., Bergström L. (2015). Nat. Nanotechnol..

[cit32] Nyström G., Marais A., Karabulut E., Wågberg L., Cui Y., Hamedi M. M. (2015). Nat. Commun..

[cit33] Sehaqui H., Gálvez M. E., Becatinni V., Cheng Ng Y., Steinfeld A., Zimmermann T., Tingaut P. (2015). Environ. Sci. Technol..

[cit34] Svagan A. J., Azizi Samir M. A. S., Berglund L. A. (2007). Biomacromolecules.

[cit35] Lee J., Deng Y. (2011). Soft Matter.

[cit36] Iwamoto S., Kai W., Isogai A., Iwata T. (2009). Biomacromolecules.

[cit37] Klemm D., Kramer F., Moritz S., Lindström T., Ankerfors M., Gray D., Dorris A. (2011). Angew. Chem., Int. Ed..

[cit38] Pääkko M., Ankerfors M., Kosonen H., Nykanen A., Ahola S., Österberg M., Ruokolainen J., Laine J., Larsson P. T., Ikkala O., Lindström T. (2007). Biomacromolecules.

[cit39] Henriksson M., Henriksson G., Berglund L. A., Lindström T. (2007). Eur. Polym. J..

[cit40] Saito T., Kimura S., Nishiyama Y., Isogai A. (2007). Biomacromolecules.

[cit41] Cervin N., Aulin C., Larsson P., Wågberg L. (2012). Cellulose.

[cit42] Cai J., Liu S., Feng J., Kimura S., Wada M., Kuga S., Zhang L. (2012). Angew. Chem., Int. Ed..

[cit43] Shi Z., Phillips G. O., Yang G. (2013). Nanoscale.

[cit44] Zhou S., Wang M., Chen X., Xu F. (2015). ACS Sustainable Chem. Eng..

[cit45] Khan Z. U., Edberg J., Hamedi M. M., Gabrielsson R., Granberg H., Wågberg L., Engquist I., Berggren M., Crispin X. (2016). Adv. Mater..

[cit46] Ali Z. M., Gibson L. J. (2013). Soft Matter.

[cit47] Svagan A. J., Berglund L. A., Jensen P. (2011). ACS Appl. Mater. Interfaces.

[cit48] Rakov E. G., Anoshkin I. V., Khung N., Saraev P. V., Malykh A. V., Nguen Man' T., Shinshin A. S., Gladkova M. P., Dubas A. L., Pozin S. I. (2008). Theor. Found. Chem. Eng..

[cit49] Hung N. C., Anoshkin I. V., Dementev A. P., Katorov D. V., Rakov E. G. (2008). Inorg. Mater..

[cit50] Zou J., Liu J., Karakoti A. S., Kumar A., Joung D., Li Q., Khondaker S. I., Seal S., Zhai L. (2010). ACS Nano.

[cit51] Pan L., Chortos A., Yu G., Wang Y., Isaacson S., Allen R., Shi Y., Dauskardt R., Bao Z. (2014). Nat. Commun..

