# Supplementary material for: Electrical behaviour of native cellulose nanofibril/carbon nanotube hybrid aerogels under cyclic compression
Source: RSC Adv. 2016 Sep 5;6(92):89051–6. doi: 10.1039/c6ra16202a (PMC5361171; doi:10.1039/c6ra16202a)
Supplement: Supplementary file 1 [file RA-006-C6RA16202A-s001.pdf]

Electronic Supplementary Material (ESI) for RSC Advances.
This journal is © The Royal Society of Chemistry 2016

Supporting Information

Electrical behaviour of native cellulose nanofibril/carbon nanotube hybrid aerogels under cyclic compression

M. Wang,[a] Ilya V. Anoshkin,[a] Albert G. Nasibulin,[a, b, c] Robin H. A. Ras,[a] Nonappa,[a] Janne Laine,[d] Esko I. Kauppinen,[a] and Olli Ikkala[*, a]

[a]Department of Applied Physics, School of Science, Aalto University,

P.O.Box 15100, FI-00076 Espoo, Finland.

[b]Skolkovo Insititute of Science and Technology, Nobel str. 3, Moscow, 143026, Russia

[c]Saint-Petersburg State Polytechnical University, Department of Material Science,

Polytechnicheskaya 29, 195251, Saint-Petersburg, Russia

[d]Department of Forest Products Technology, School of Chemical Technology,

Aalto University, P.O.Box 16300, FI-00076, Espoo, Finland.

E-mail:olli.ikkala@aalto.fi

Figure S1. Transmission electron microscopy images of FWCNTs dispersed in water.

Figure S2. Scanning electron microscopy images of pristine CNF aerogel and FWCNT/CNF 15/85 wt/wt aerogel.

Figure S3. Cyclic mechanical and electrical compression tests for FWCNT/CNF 25/75 wt/wt (a-d) and FWCNT/CNF 15/85 wt/wt aerogels (e-f).

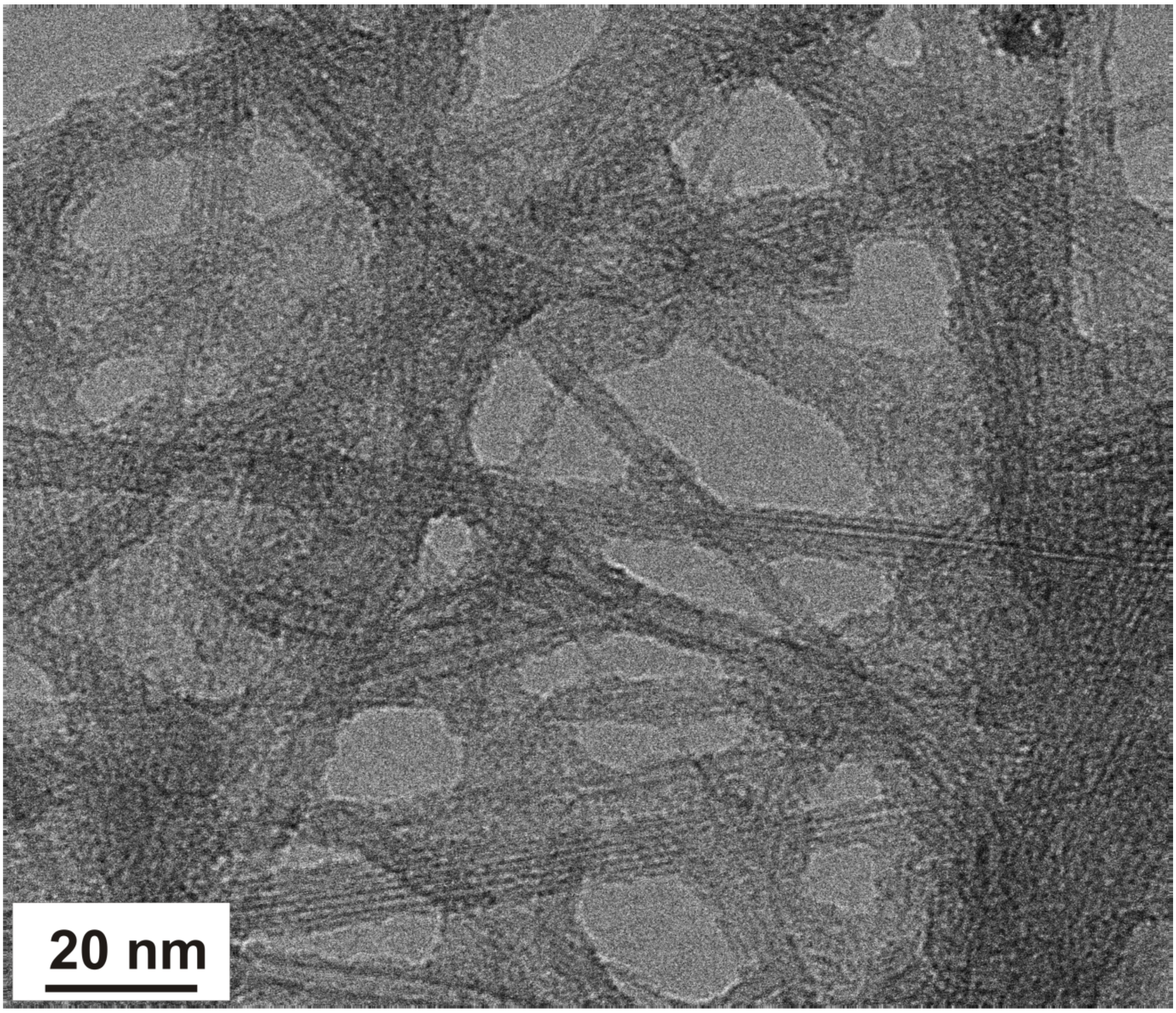

Figure S1. Transmission electron microscopy image of dispersed FWCNT in water from a diluted suspension. It shows individualized FWCNT's diameter of 3.2 nm, as well as their bundles.

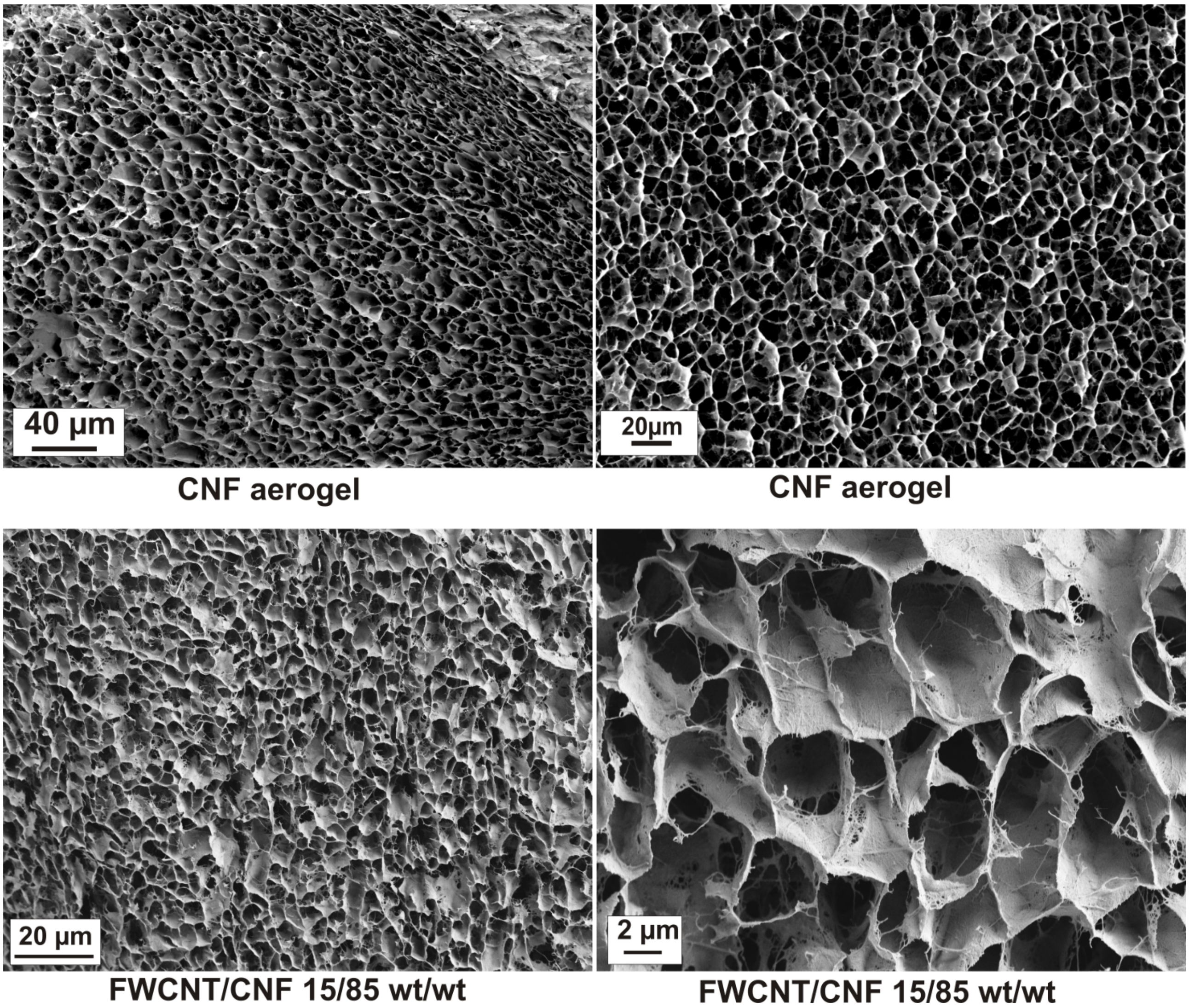

Figure S2. Scanning electron microscopy images of pristine CNF aerogel and FWCNT/CNF 15/85 wt/wt aerogels. The images show cellular structure without and with CNT in the networks.

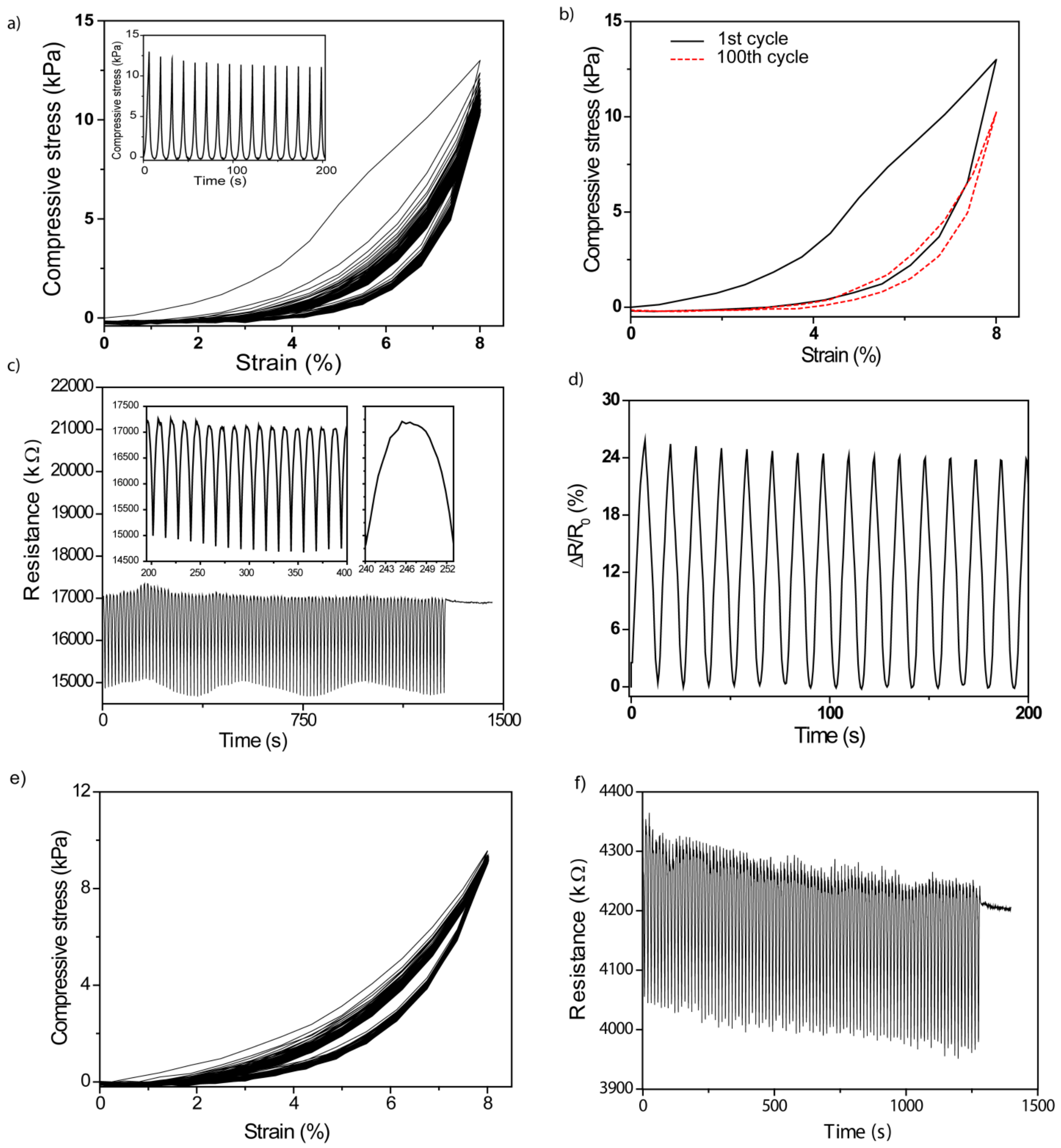

Fig S3. (a) 100 cycled stress-strain curves up to strain 8% of FWCNT/CNF 25/75 wt/wt aerogel and the 16 first cycles compressive stress *vs*. time illustrated (inset). (b) The first and 100th cycles of compressive stress-strain curves of FWCNT/CNF 25/75 wt/wt aerogel. (c) 100 cycled resistance response *vs*. time under cyclic compression up to strain 8% of FWCNT/CNF 25/75 wt/wt aerogel. The insets show the 16 first cycles. (d) Fractional resistance reduction during cyclic compression of FWCNT/CNF 25/75 wt/wt aerogel. (e) 100 cycled stress-strain curves up to strain 8% of FWCNT/CNF 15/85 wt/wt aerogel. (f) 100 cycled resistance *vs*. time under cyclic compression up to strain 8% of FWCNT/CNF 15/85 wt/wt aerogel. Resistance changes irreversibly of FWCNT/CNF 15/85 wt/wt aerogel upon loading and unloading. It gradually decreases under cyclic compression.
